# Supplementary material for: Identification of photoperiod-induced specific miRNAs in the adrenal glands of Sunite sheep (Ovis aries)
Source: Front Vet Sci. 2022 Jul 22;9:888207. doi: 10.3389/fvets.2022.888207 (PMC9354845; doi:10.3389/fvets.2022.888207)
Supplement: Supplementary file 1 [file Data_Sheet_1.ZIP › Supplementary file/miRDeep2 file.zip!/miRDeep2 file/Novel_Structure/LP42A1/Novel_247.pdf]

Provisional ID	:	NC_019463.2_12977
Score total	:	2.3
Score for star read(s)	:	-1.3
Score for read counts	:	0
Score for mfe	:	2
Score for randfold	:	1.6
Score for cons. seed	:	
Total read count	:	213
Mature read count	:	213
Loop read count	:	0
Star read count	:	0

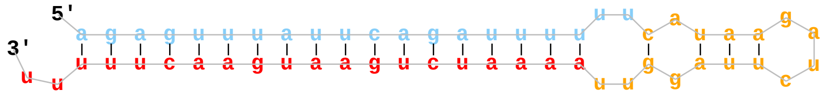

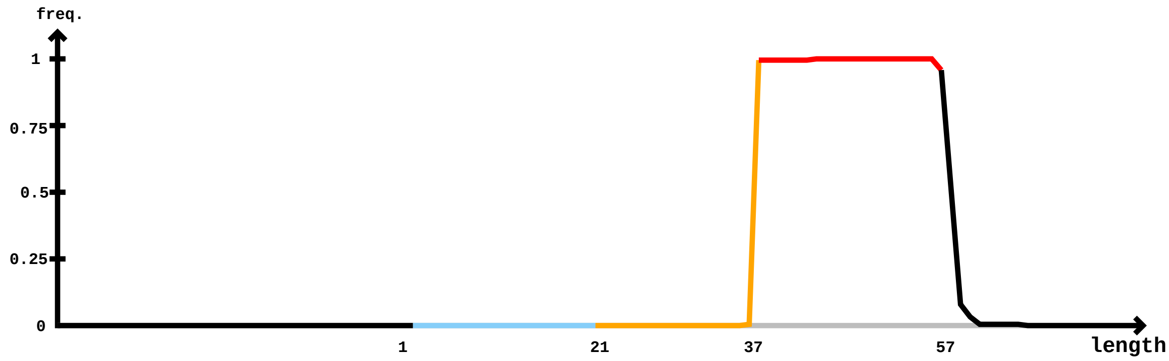

Star Mature

```
5'- uacuauuaacucuaaauaguuaauagguuggccaaagaguuuauucagauuuuuucauaagaucuuagguuaaaaucugaaugaacuuuuuggccaaguuacuaauuuaau   -3'   exp
    ..(((((((((......))))))))).((((((((((((((((((((((((((..(.(((....))).)..))))))))))))))))))))))))))..............  reads   mm   sample
    ......................................................................uaaaaucugaaugaacuuuuu....................  1       0    seq
    .......................................................................aaaaucugaaugaacuuuu.....................  9       0    seq
    .......................................................................aaaaucugaaugaacuuuuA....................  6       1    seq
    .......................................................................aaaaucugaaCgaacuuuuu....................  8       1    seq
    .......................................................................aaaCucugaaugaacuuuuu....................  3       1    seq
    .......................................................................aaaaucugaaugaacuuuuu....................  126     0    seq
    .......................................................................aaaaucugaauAaacuuuuu....................  1       1    seq
    .......................................................................aaaaucAgaaugaacuuuuu....................  1       1    seq
    .......................................................................aaaaucugaaugaacuuuuug...................  36      0    seq
    .......................................................................aaaaucugaaugaacuuuuuU...................  6       1    seq
    .......................................................................aaaaucugaaCgaacuuuuugg..................  2       1    seq
    .......................................................................aaaaucugaaugaacCuuuugg..................  1       1    seq
    .......................................................................aaaaAcugaaugaacuuuuugg..................  1       1    seq
    .......................................................................aaaCucugaaugaacuuuuugg..................  6       1    seq
    .......................................................................aaaaucugaaugaacuuuuuggc.................  4       0    seq
    .......................................................................aaaCucugaaugaacuuuuuggc.................  2       1    seq
    .............................................................................ugaaugaacuuuuuggccaagC............  1       1    seq
```
